# Mathematical Modeling of Tuberculosis Granuloma Activation

**DOI:** 10.3390/pr5040079

**Published:** 2017-12-11

**Authors:** Steve M. Ruggiero, Minu R. Pilvankar, Ashlee N. Ford Versypt

**Affiliations:** 1 School of Chemical Engineering, Oklahoma State University, Stillwater, OK 74078, USA;; 2 Oklahoma Center for Respiratory and Infectious Diseases, Oklahoma State University, Stillwater, OK 74078, USA

**Keywords:** latent tuberculosis, immune system, cytokine signaling network, dynamic systems, collagen remodeling

## Abstract

Tuberculosis (TB) is one of the most common infectious diseases worldwide. It is estimated that one-third of the world’s population is infected with TB. Most have the latent stage of the disease that can later transition to active TB disease. TB is spread by aerosol droplets containing Mycobacterium tuberculosis (Mtb). Mtb bacteria enter through the respiratory system and are attacked by the immune system in the lungs. The bacteria are clustered and contained by macrophages into cellular aggregates called granulomas. These granulomas can hold the bacteria dormant for long periods of time in latent TB. The bacteria can be perturbed from latency to active TB disease in a process called granuloma activation when the granulomas are compromised by other immune response events in a host, such as HIV, cancer, or aging. Dysregulation of matrix metalloproteinase 1 (MMP-1) has been recently implicated in granuloma activation through experimental studies, but the mechanism is not well understood. Animal and human studies currently cannot probe the dynamics of activation, so a computational model is developed to fill this gap. This dynamic mathematical model focuses specifically on the latent to active transition after the initial immune response has successfully formed a granuloma. Bacterial leakage from latent granulomas is successfully simulated in response to the MMP-1 dynamics under several scenarios for granuloma activation.

## 1. Introduction

Tuberculosis (TB) has killed more people than any other infectious disease and continues to infect more people today than at any other time in history [1]. In 2015, 10.4 million people were infected with Mycobacterium tuberculosis (Mtb), and 1.8 million died from TB disease [2]. An individual inoculated with Mtb may experience a range of outcomes. The Mtb bacteria may be immediately destroyed by the host’s immune response, the immune response may isolate bacteria into granulomas where the infection persists in a latent state, or the bacteria may proliferate and manifest as active TB disease if the initial infection is not controlled by the immune response. A majority of people infected with Mtb have a clinically latent infection in which they do not show any symptoms of the infection. These individuals serve as a reservoir for the bacteria, and if their immune response system is compromised in such a way to trigger the penetration of the granulomas by active bacteria and formation of TB cavities, the infection may transition from latent to active TB disease. The major risk factors for activation of TB after an extended latent period include contact with an infectious TB patient, HIV co-infection, initiation of an anti-tumor necrosis factor (TNF) treatment, silicosis, and diabetes [3]. About 5–10% of latent infections undergo granuloma activation and progress to active TB [4]. However, the mechanism for the activation of latent TB is still unclear. Improved understanding of the triggers and dynamics of this transition could be useful for designing new therapies to prevent the activation of latent TB.

**Figure 1.**
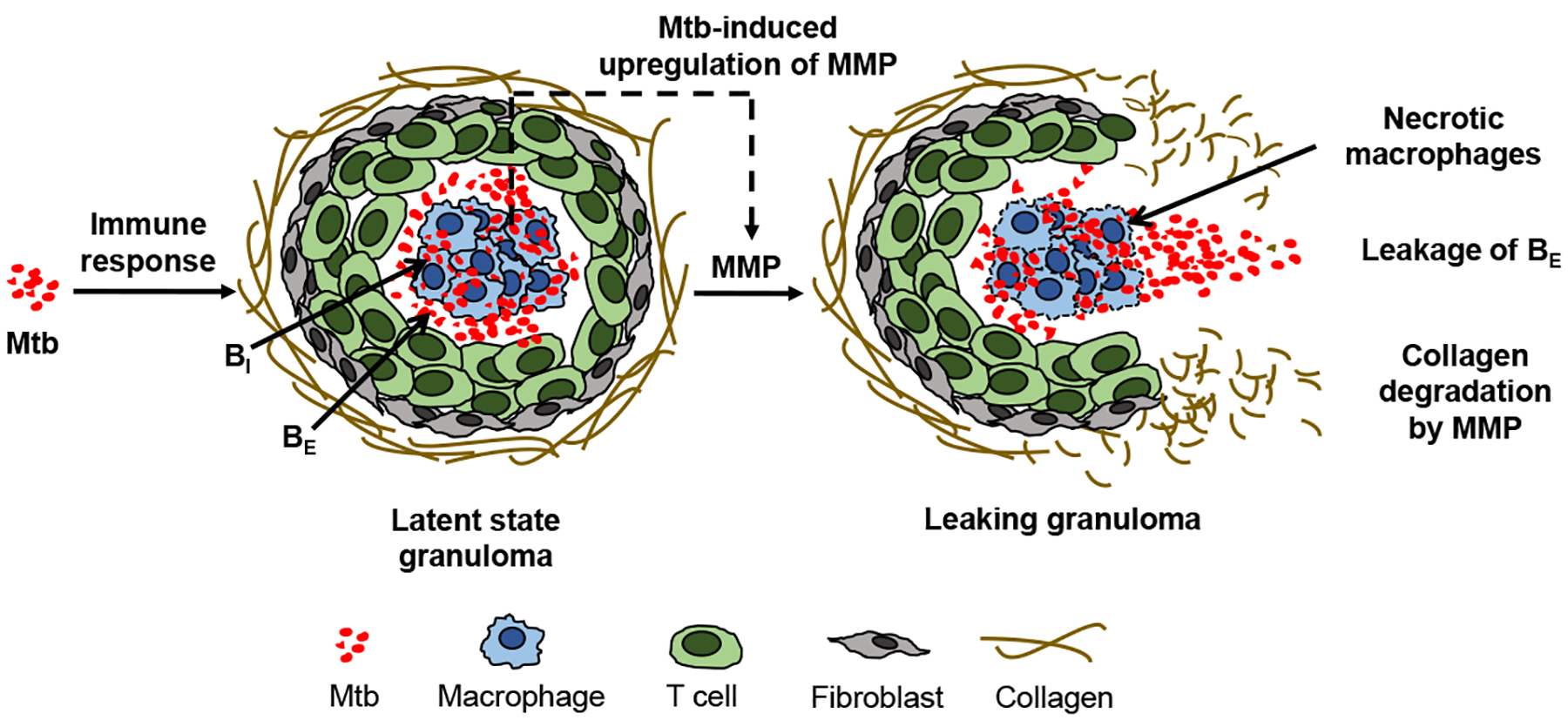
Mycobacterium tuberculosis (Mtb) induces an immune response in the lungs of a host that can lead to formation of a cellular aggregate called a granuloma in which the Mtb can remain dormant in the condition of latent tuberculosis (TB). Direct and indirect upregulation of matrix metalloproteinase (MMP), which is stimulated by the Mtb in infected macrophages in the granuloma and denoted by a dashed arrow, degrades the collagen exterior of the granuloma, triggering leakage of extracellular bacteria (*B*_*E*_) and formation of a necrotic leaking granuloma called a TB cavity.

TB infection starts when infectious droplets containing Mtb reach the respiratory tract of an individual. After reaching the lung tissue, the Mtb is ingested by the resident alveolar macrophages. The host cellular immune response starts with the secretion of cytokines, such as interleukin 12 (IL-12) and tumor necrosis factor alpha (TNF-*α*), and chemokines that recruit the immune cells to the site of infection [5] to form a compact cluster of immune cells, known as a granuloma. Latent infection is characterized by granuloma formation and steady state maintenance (Figure 1). A granuloma is mostly comprised of an organized aggregate of blood-derived macrophages that ingest and contain the bacteria, differentiated macrophages, and T cells along with other cells such as neutrophils, multinucleated giant cells, dendritic cells, B cells, natural killer cells, fibroblasts, and cells that secrete extracellular matrix components. The exterior surface of the granuloma is composed largely of collagen fibers (Figure 1). The granuloma acts as a microenvironment that walls off the bacteria from the rest of the body to control the infection [1].

Collagen fibers provide tensile strength to the lungs and to granulomas and are highly resistant to enzymatic degradation. Only collagenolytic proteases such as matrix metalloproteinases (MMPs) are able to break the collagen fibers that encapsulate a granuloma. MMPs are a family of proteolytic enzymes that degrade the components of the extracellular matrix and are critical for matrix remodeling [6]. MMPs are typically regulated by the complementary class of inhibitors called tissue inhibitors of metalloproteinases (TIMPs). MMPs have been implicated in the activation of latent tuberculosis infections [7–9]. From the MMP family of proteases, MMP-1 specifically degrades type-1 collagen and drives the remodeling of pulmonary tissue in TB [7]. Experimental data showed that TB activation involved a dysregulation in the balance of MMP-1 and its inhibitor TIMP-1 [7,10]. Direct infection of macrophages induced gene expression and secretion of MMP-1 along with a few other MMPs [7]. Additionally, pro-inflammatory cytokines increased MMP secretion from stromal cells such as epithelial cells, fibroblasts, and astrocytes, while there was no compensatory increase in the production of TIMPs to regulate the MMP levels, thus causing an increase in collagen degradation [7]. Measurements from both human plasma samples [10] and sputum [7] showed that the concentrations of MMP-1 were elevated in patients with active TB compared to latent TB patients or non-infected control subjects, and the levels of the associated inhibitor TIMP-1 were either decreased [7] or changed insignificantly [10] in the active TB cohorts. In [7] MMP-1, degradation of lung collagen in TB was confirmed using a transgenic TB mouse model that overexpressed human MMP-1. As MMP-1 is the dominant MMP in granuloma degradation, we simply refer to MMP-1 as MMP in the rest of this article. A surplus concentration of MMP cleaves the collagen envelope of granulomas and eventually leads to the leakage of bacteria into the airways [8] (Figure 1); from the airways, the bacteria can spread into other regions of the lung and to the rest of the patient’s body in an active infection.

The animal models that are most commonly used to study TB do not develop lung pathology exactly the same as in humans [11,12]. Mouse models are useful for studying the infection stage of TB but not the long term latency or reactivation from the latent state because most mouse models do not form human-like granulomas [7]. The rabbit model used in [10] forms necrotic, leaking cavity structures characteristic of active TB after disruption of granulomas. These cavities in rabbits are consistent with the structure observed in humans, providing a valuable animal model for mechanistic insight into cavity formation due to MMP/TIMP imbalance [10]. However, the one month time scale for the formation of active cavities from initial TB infection in the rabbit model is a much faster time scale than the typical cavity formation in human TB infection. Human studies to enhance mechanistic understanding of the untreated latent to active transition cannot be conducted ethically without applying the current standard of care (pharmaceutical interventions), disrupting the cavity formation and progress over time. Additionally, most animal models require that infected animals be sacrificed for invasive lung tissue sample collected to permit observation of the interaction between Mtb and host structures. Thus, conclusions have to be drawn at minimal discrete time points without providing much insight into the dynamic processes. Another surrogate model system is needed to overcome these challenges for understanding the dynamics of MMP dysregulation that can lead to TB cavity formation from latent granulomas and reactivation to TB disease from latent disease.

In lieu of biological experiments, computational models can be used to test possible mechanisms for triggering the switch from latent to active TB infection as well as to study the dynamic process. Mathematical models are useful tools for inexpensively conducting in silico experiments with multiple interacting factors and for testing hypotheses. We developed a mathematical model in this study to probe triggers for inducing the latent to active transition. Several computational and mathematical models have been developed to describe the granuloma formation stages of TB in response to an initial infection [13–16]. Another model was used to explore activation of TB due to a pharmaceutical intervention [17]. No mathematical model has been published addressing the impact of MMP dysregulation or the dynamics of this process on the biological network of cells within a granuloma during TB. The model developed here builds on an existing model of the immune response to Mtb [13] (referred to as the “immune response model” henceforth) by extending this model to explicitly consider dynamic regulation of MMP-1. We also share our open-source Python codes for ease of continued development by other computational researchers and by expanding the horizons of use of the models for further in silico experiments by collaborators and other scientists not necessarily trained in high performance computing.

The immune response model is able to simulate three physiologically-relevant regimes based on parameter values: (i) immediate clearance of Mtb; (ii) a mild initial infection followed by long-term latent TB; and (iii) an initial uncontrolled active infection [13]. Here, a novel model for MMP dynamics, collagen degradation, and bacterial leakage is added to the immune response model. Figure 1 illustrates the mechanisms we aim to capture in the model. The upregulation of MMP by Mtb drives the degradation of the granuloma envelope, which allows Mtb to leak out of the granuloma to the surroundings. Using the model that incorporates the MMP dynamics, we investigated conditions under which the biological system can be perturbed to switch to active infection after a steady latent infection has been established. Section 2 details the equations used to define the mathematical model. Section 3 includes model results under various scenarios as well as an analysis of the model sensitivity to parameter values.

## 2. Methods

The immune response model considers the local immune response to Mtb in the lungs. The immune response model includes population balances for macrophages, two families of T cells (CD4+ and CD8+), intracellular bacteria (inside infected macrophages), and extracellular bacteria (inside the granuloma but outside the macrophages). The model also includes the signaling network that connects the various cell populations via the cytokines TNF-*α*, interferon gamma (IFN-*γ*), IL-4, IL-10, and IL-12 [13]. The equations and parameters of the immune response model are summarized in the Supplementary Materials. The immune response model can generate three regimes representing the infection outcomes of clearance, latency, and active TB. However, the immune response model does not consider the dynamic effects of MMP on the collagen on the surface of the granuloma, which, if breached, can lead to bacterial leakage. In addition, the immune response model cannot predict the transition from latent to active TB after a period of latency. The present work extends the immune response model by considering the effects of intracellular bacteria on reprogramming infected macrophages to increase production of MMP and the subsequent degradation of the collagen envelope of granuloma by MMP. Here, we focus on additions to the original immune response model that represent the local changes in MMP concentration, collagen concentration, and the leakage of extracellular bacteria from the granuloma. Each of these additions is described in turn in the following subsections. Here, the granulomas are considered as well-mixed zones without transport limitations to facilitate adaptation of the ordinary differential equation based immune response model. An alternate partial differential equation model for the immune response with spatial effects including diffusion has been formulated [16]. However, the simulation results for that model were only shown for spatially averaged populations of cells and cytokines. We seek to improve understanding of the process dynamics in the present work and thus follow these previously published models in neglecting spatial effects inside of granulomas.

### 2.1. MMP Dynamics

**Figure 2.**
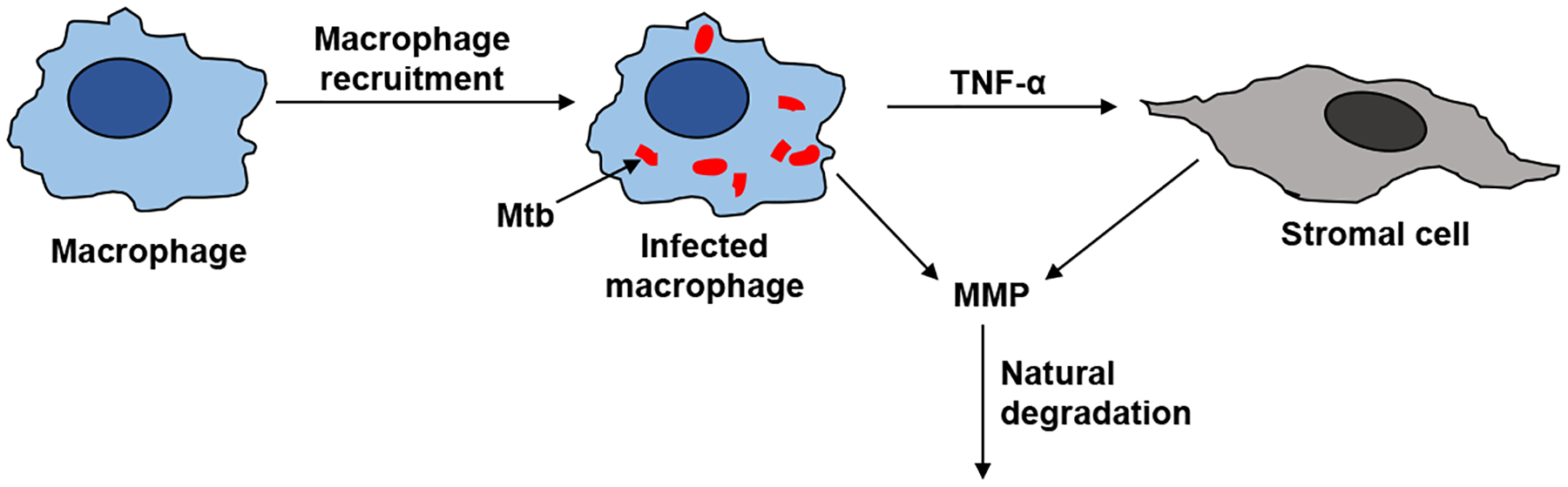
Resting macrophages recruit active macrophages in the immune response. Mtb infects some of the macrophages. The infected macrophages can upregulate MMP secretion directly (denoted by arrow from infected macrophage to MMP) and indirectly via TNF-*α* signaling to stromal cells (indicated by the arrows connecting the stromal cell to the infected macrophage and MMP). Tissue inhibitors of metalloproteinase (TIMP) is not correspondingly upregulated to inhibit MMP, making the enzyme’s degradation rate the primary consumption term for surplus MMP that basal TIMP can not regulate.

The steps that affect the MMP dynamics are illustrated in Figure 2. The activation of resting macrophages, the recruitment of additional macrophages, and the infection of macrophages by Mtb are well-characterized by the immune response model. Macrophages infected with Mtb have been observed to induce gene expression and secretion of MMP; however, a compensatory increase in secretion of TIMP was not observed [7]. The infected macrophages are not the only source of MMP in a granuloma. The infected macrophages interact with the stromal cells like epithelial cells, fibroblasts, and astrocytes, which further secrete MMP and together amplify the MMP upregulation. The pro-inflammatory cytokines especially TNF-*α* have been found to play a key role in triggering the upregulation of MMPs by stromal cells [18]. It has been found that interaction between macrophages and stromal cell requires TNF-*α* to increase the MMP secretion by stromal cell networks [18–21].

The mass balance for MMP in terms of concentration for a constant volume system, [MMP], is

(1) $$\frac{d[\text{MMP}]}{dt}=\alpha_{\text{MMP}}M_{I}\frac{F_{\alpha }}{F_{\alpha }+s_{\text{MMP}}}+\beta_{\text{MMP}}M_{I}-\mu_{\text{MMP}}[\text{MMP}]+sr_{\text{MMP}},$$

where the first term represents secretion of MMPs indirectly by the stromal cells that requires both TNF-*α*, *F*_*α*_, and infected macrophages, *M*_*I*_; the second term represents production of MMPs by reprogrammed infected macrophages; the last two terms represent the natural first-order degradation and constant production of MMP; *α*_MMP_ is the rate constant for indirect production of MMP; *s*_MMP_ is the constant where the effect of TNF-*α* on the indirect MMP production has reached half of its saturation level; *β*_MMP_ is the rate constant for direct production of MMP by *M*_*I*_; *μ*_MMP_ is the half life of MMP; and *sr*_MMP_ is the basal constant recruitment rate of MMP. The last two terms maintain the constant concentration of MMP at equilibrium in the latent state. The functional forms for the two terms representing the upregulation of MMP in the presence of Mtb infection were based on the general mathematical forms defined in [13]: i.e., all terms that require the presence of infected macrophages to upregulate a process are given a linear dependence on *M*_*I*_, while all terms that are upregulated by a cytokine such as TNF-*α* are given a Michaelis-Menten type saturation equation dependent on *F*_*α*_.

### 2.2. Collagen Dynamics

**Figure 3.**
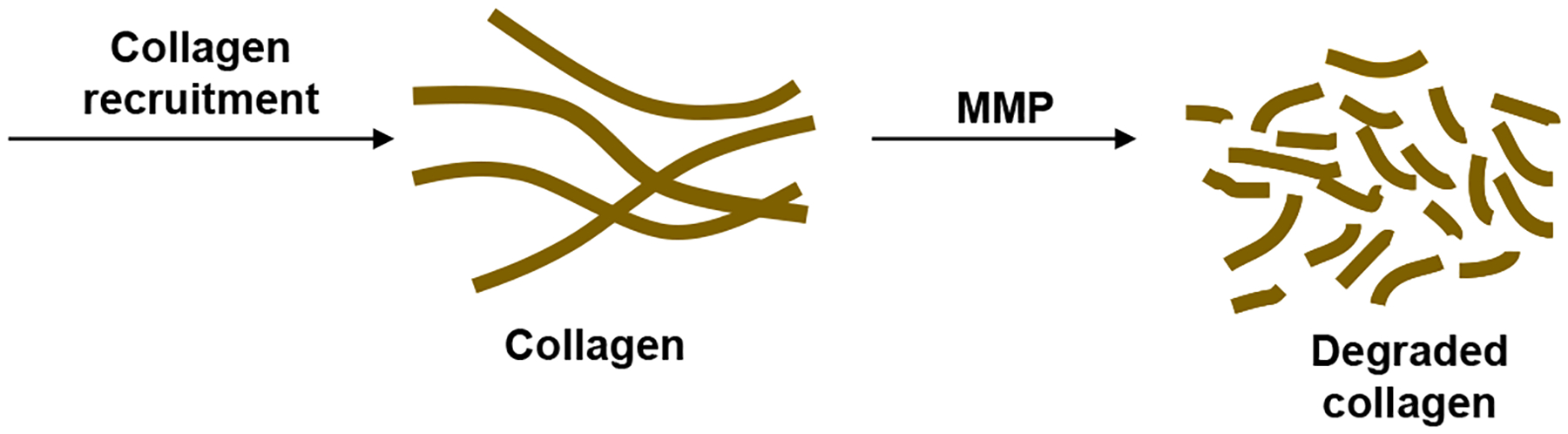
The initial formation of granulomas involves collagen recruitment to form the fibrillar collagen network of the stable granulomas in latent TB. Upregulation of MMP degrades the collagen making the granulomas penetrable by Mtb.

The collagen dynamics during granuloma formation at the onset of TB infection is beyond the scope of the current work focused on the latent to active transition. Therefore, for simplicity, we consider a constant source term for collagen representative of the source after latency is achieved. The effects of MMP on degrading the collagen are incorporated to study how the granulomas can be compromised after latent TB is established (Figure 3). The cleavage of collagen by MMPs was found to display Michaelis–Menten kinetics [22,23]. We recognize that the granulomas should have the collagen fibers concentrated on the exterior surfaces. In the model proposed here, there is no spatial variation. This could be a realistic approximation if the MMP is uniformly secreted within and adjacent to the granulomas or if the transport occurs faster than the degradation time scale. Slow collagen degradation is considered here, making this well-mixed model reasonable. The mass balance for the change in concentration of collagen, *C*, in the well-mixed granuloma is

(2) $$\frac{dC}{dt}=sr_{C}-k_{C}[\text{MMP}]\frac{C}{C+k_{M}},$$

where *sr*_*C*_ is a constant recruitment term representing the external build up of the collagen envelope around the granuloma, *k*_*C*_ is the rate constant of collagen degradation, and *k*_*M*_ is the Michaelis constant for collagen degradation catalyzed by MMP.

### 2.3. Bacterial Leakage

The change in the population of the extracellular bacteria inside the granuloma, *B*_*E*_, has two terms:

(3) $$\frac{dB_{E}}{dt}=\frac{dB_{E,IR}}{dt}+\frac{dB_{E,L}}{dt},$$

where *B*_*E*,*IR*_ is the non-leaking extracullular bacteria and *B*_*E*,*L*_ is the leaking extracellular bacteria (Figure 1). The first term $\frac{dB_{E,IR}}{dt}$ is the contribution from the immune response model and is given by (S16) in the Supplementary Materials, which considers all the different mechanisms for the gain and loss in extracellular bacterial count corresponding to the release and uptake of intracellular bacteria by the macrophages, respectively, and the constant turnover number. However, this term does not capture the loss of extracellular bacteria population when the granuloma starts leaking extracellular bacteria into the lung. To account for this case, the second term is added to (3) to track the leakage of bacteria through deteriorated collagen and is represented by

(4) $$\frac{dB_{E,L}}{dt}=-k_{L}s_{L}B_{E}\frac{1-\frac{C}{C_{La}}}{C+s_{L}},$$

where *k*_*L*_ is the rate constant for the maximum rate of bacteria exiting the granuloma, *s*_*L*_ is the half saturation constant for the inhibitory effect of collagen on this process, and *C*_*La*_ is the expected collagen concentration at latency. When the concentration of collagen is equal to the concentration of collagen in the latent case (*C* = *C*_*La*_), the granuloma is intact with no leakage of bacteria, thus making the leakage term (4) zero. Zero is the maximum value for (4), i.e., the bacterial leakage never has a positive value. This is ensured by the maximum value of *C* in the model formulation, which is *C*_*La*_. The maximum collagen concentration with respect to changes in MMP concentration is determined by $\frac{\partial C}{\partial [\text{MMP}]}=\frac{\partial C}{\partial t}/\frac{\partial [\text{MMP}]}{\partial t}=0$. The solution to the maximization problem is $sr_{C}=k_{C}[\text{MMP}]\frac{C}{C+k_{M}}$, which is how *sr*_*C*_ was defined using the values [MMP] = [MMP]_*La*_ and *C* = *C*_*La*_. At the other extreme, when the concentration of collagen goes to zero, there is no longer a barrier around the bacteria, and the rate of leakage is directly dependent on the extracellular bacterial count giving the fastest bacteria leakage rate from the granuloma.

### 2.4. Biological Feedback

Although not shown explicitly, a feedback loop is formed between the equations introduced here (1)–(4) via the species included in the immune response model (see Supplementary Materials (S1)–(S16)). It is apparent that Equation (2) depends on the value of (MMP), and Equations (3)–(4) depend on *C*. The contribution to the extracellular bacterial count from the immune response *B*_*E*,*IR*_ depends on multiple species in (S16) including *B*_*E*_. The extracellular bacteria count *B*_*E*_ in-turn leads directly to changes in species *M*_*R*_, *M*_*I*_, *M*_*A*_, *F*_*α*_, *I*_*γ*_, *I*_12_, and *B*_*I*_ through dependence on *B*_*E*_ or *B*_*T*_ = *B*_*E*_ + *B*_*I*_ in equations (S1), (S2), (S3), (S10), (S11), (S14), and (S15), respectively. Furthermore, changes in *M*_*I*_, *M*_*A*_, *I*_*γ*_, *B*_*T*_, and some T cells affect the production of *F*_*α*_. Changes in *M*_*I*_ and *F*_*α*_ directly lead to changes in the production of MMP given by (1). Other pathways for indirect feedback exist between the cytokines and the bacterial-population-sensitive macrophages.

### 2.5. Parameter Values

Parameters need to be specified to define the system before performing any simulations. A value of *μ*_MMP_ is taken from a mathematical model for MMP in fibrosis [24] and is used as the basis for calculating the rest of the parameters for (1). The value of *sr*_MMP_ is calculated by evaluating (1) with no infection and data from [25]. The value of *F*_*a*_ at the end of a typical latent simulation is used to calculate *s*_MMP_. Values of *α*_MMP_ and *β*_MMP_ are then calculated using data from [7,25]. Both *k*_*C*_ and *k*_*M*_ are kinetic parameters taken from published experimental data on characterizing the kinetics of MMP. However, the parameters for the breakdown of collagen in literature have two sets of parameters: one for each of the two proteins, *α*−1 and *α*−2, that compose collagen I [23]. For this model, a weighted average of the parameters based on the number of proteins in each stand is used and converted into the appropriate units. The source term for collagen I, *sr*_*C*_, is then calculated from a typical collagen concentration [24]. The value of collagen expected in the latent case, *C*_*La*_, is set based on data from the same fibrosis model used for *μ*_MMP_ [24]. The rest of the parameters in (4), *s*_*C*_, *k*_*L*_, and *s*_*L*_, are calculated to give the steady state and reasonable limiting behavior. The parameters are defined in Table 1. The parameters listed in the Supplementary Materials Table S1 were validated for the immune response model [13].

### 2.6. Numerical Methods and Code Repository

The system of ordinary differential equations in the immune response model (see Supplementary Materials (S1)–(S16)) and the TB granuloma active model defined by (1)–(4) was solved with odeint solver from the SciPy Integrate Python module, which uses the classic lsoda routine from the FORTAN library odepack. The default options were used in the solver. The parameter values in Table 1 were used to generate the results in Section 3, unless otherwise indicated. The initial conditions are given in Table 2. To enable code reuse, we wrote the model in Python and shared the code, parameter files, and documentation in an open-source software repository at http://github.com/ashleefv/tbActivationDynamics [26].

## 3. Results and Discussion

### 3.1. Representative Latent Case

**Figure 4.**
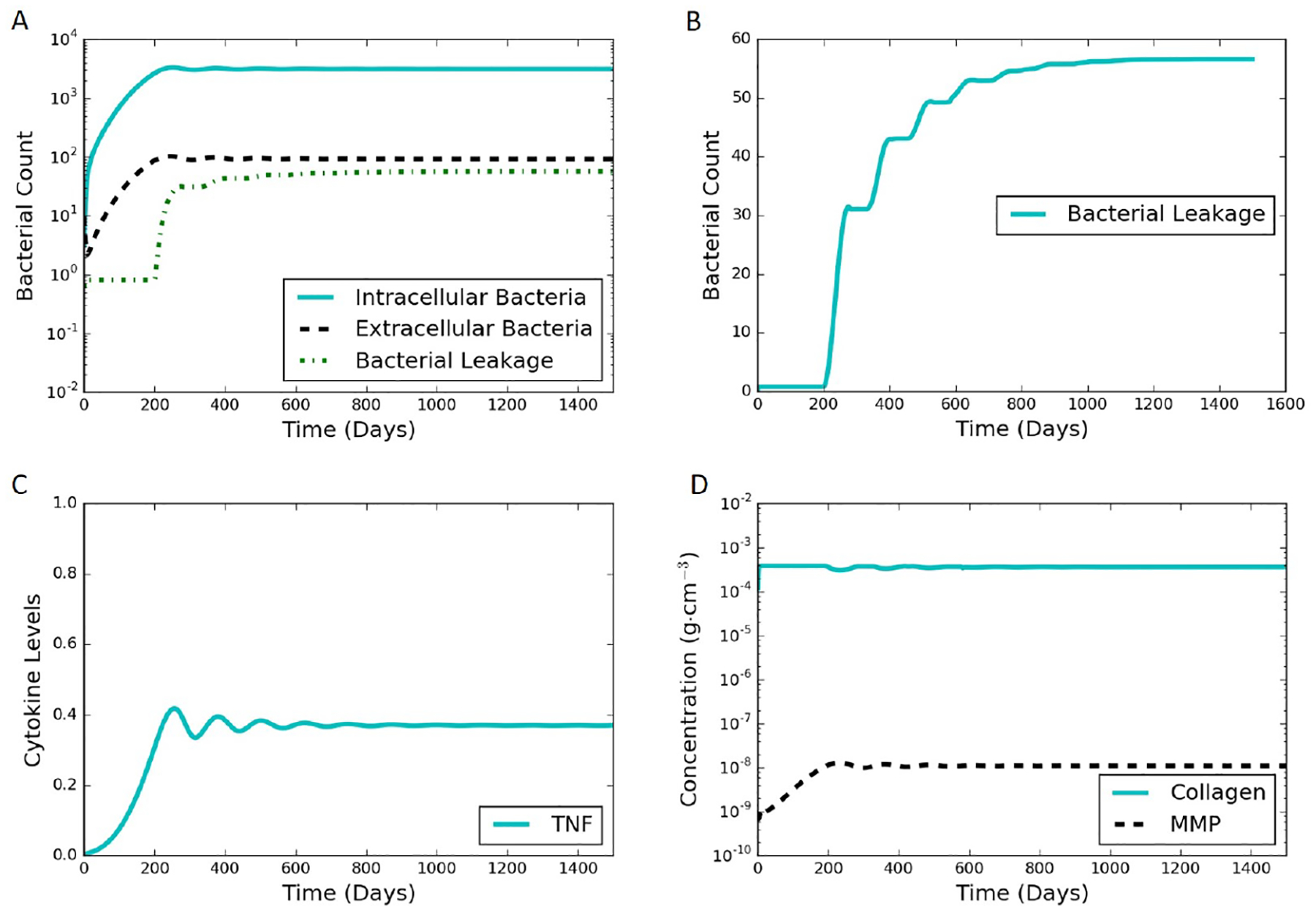
Typical simulation results leading to latent infection. All the concentrations and populations stabilize over time, indicating latency. (**A**) bacterial populations and cumulative bacterial leakage (log scale) vs. time. The intracellular, extracellular, and leaked bacterial concentrations are successfully controlled by the immune system around 200 days; (**B**) cumulative bacterial leakage (linear scale) vs. time; (**C**) dimensionless concentration of cytokine tumor necrosis factor (TNF)-*α* (linear scale) vs. time; (**D**) concentrations of MMP and collagen vs. time on a log scale.

The model proposed in Section 2 is able to simulate both latent and active infections depending on the different parameter values selected. We used substantial leakage of extracellular bacteria as the marker for the transition from latent to active infection. Figure 4 shows model results of a representative latent case using the parameter values listed in Table 1 and Table S1 in the Supplementary Materials. This latent case is also leaking bacteria over time with a very small but nonzero amount of bacteria escaping the granuloma. The leaking case eventually stabilizes and tends to a steady state without further bacterial leakage. It should be noted that the cumulative bacterial leakage observed in Figure 4B after 200 days is on a linear scale compared to the log scale in Figure 4A and is not significant compared to the total bacterial count inside the granuloma. Intracellular bacteria are the bacteria inside the infected macrophages. These infected macrophages secrete TNF-*α*. The MMP increase tracks with the TNF-*α* increase, except that the oscillations in MMP are damped compared to those for TNF-*α*. Around 200 days, the MMP concentration passes a threshold that starts to degrade collagen causing the bacteria leakage term to start growing. The entire system starts stabilizing after that due to the feedback processes, eventually leading to a steady latent state.

### 3.2. Sensitivity Analysis

**Figure 5.**
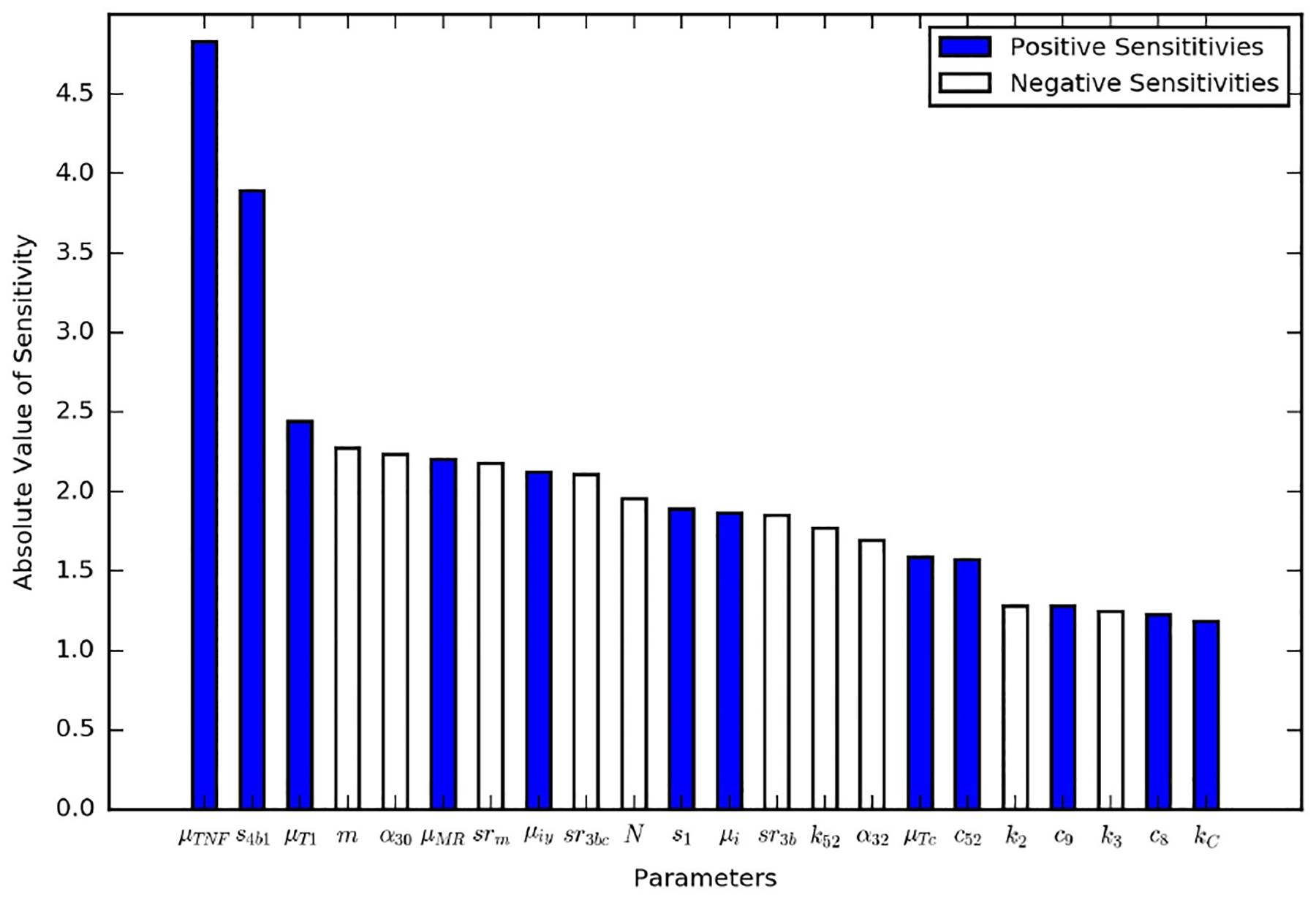
Local sensitivity analysis results for the parameters from the immune response model that were at least as sensitive as the new model parameter *k*_*C*_.

In the immune response model, the parameters were probed with a global sensitivity analysis. Here, we conducted a local sensitivity analysis on all of the new parameters for the TB granuloma activation model (Table 1) as well as the parameters for the immume response model (Table S1). The nominal set of parameters were those listed in the tables except for a *k*_*C*_ value of 2.82 × 10^5^ day^−1^, which is double the latent case value and corresponds to a leaking case. The model output of interest was the total bacterial leakage after the three years (1095 days), which is denoted as *B*_*L*_. The model output with the nominal set of parameters is *B*_*Lbase*_. All of the parameters were changed one at time by 10 scale factors, *s*_*f*_, ranging from 0.95 to 1.05 in uniform increments, i.e., increases and decreases by 1%, 2%, …, 5%. The normalized local sensitivity index, *S*_*local*_, was calculated using

(5) $$S_{\text{local}}=\frac{B_{\text{Lbase}}-B_{L}}{B_{\text{Lbase}}\left(1-s_{f}\right),}$$

which is the percent change in bacterial leakage divided by the percent change in the parameter value. Positive numbers suggest increasing the parameter increases the bacterial leakage, and negative numbers suggest decreasing the parameter increases the bacterial leakage. Figure 5 contains the results of the local sensitivity averaged for the range of tested scale factors. All of the parameters were investigated, but only those that were at least as sensitive as *k*_*C*_ are shown in Figure 5. These local sensitivity results are consistent with the global sensitivity results in [13]. *k*_*C*_ is the most sensitive of the parameters introduced here.

### 3.3. Effects of Collagen Degradation Rate Constant k_C_

**Figure 6.**
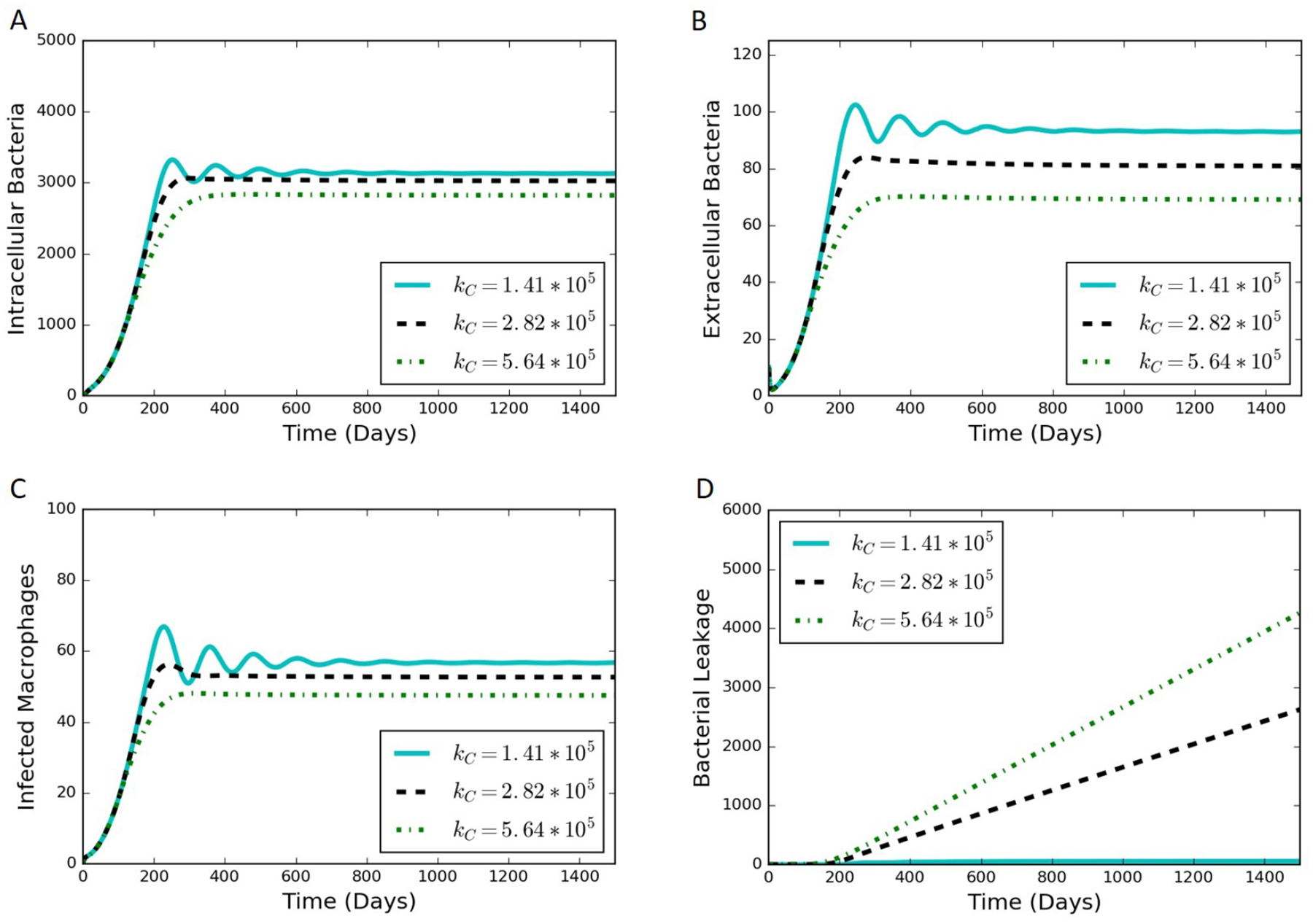
Simulation results from varying values of *k*_*C*_, where increasing *k*_*C*_ leads towards a leaking state of the granuloma. (**A**) intracellular bacteria count vs. time; (**B**) extracellular bacteria count vs. time; (**C**) infected macrophage count vs. time; (**D**) cumulative bacterial leakage vs. time.

We adjusted the rate constant of collagen degradation, *k*_*C*_, to determine the effect of the rate of leakage on the other outputs of the model. Not only is *k*_*C*_ the most sensitive of the parameters introduced in Section 2, but it is also the most uncertain of those parameters. There exists a value of *k*_*C*_ where the rate of bacterial leakage is zero, and increasing *k*_*C*_ only affects the other variables of the model through a reduction in collagen allowing more bacteria to leak. Figure 6 contains simulation results at various values of *k*_*C*_. Increases in *k*_*C*_ have a dampening effect on the oscillations observed in the system (Figure 6A–C) and can lead to a substantial increase in bacterial leakage (Figure 6D).

### 3.4. In Silico Experiment Perturbing the Immune System

**Figure 7.**
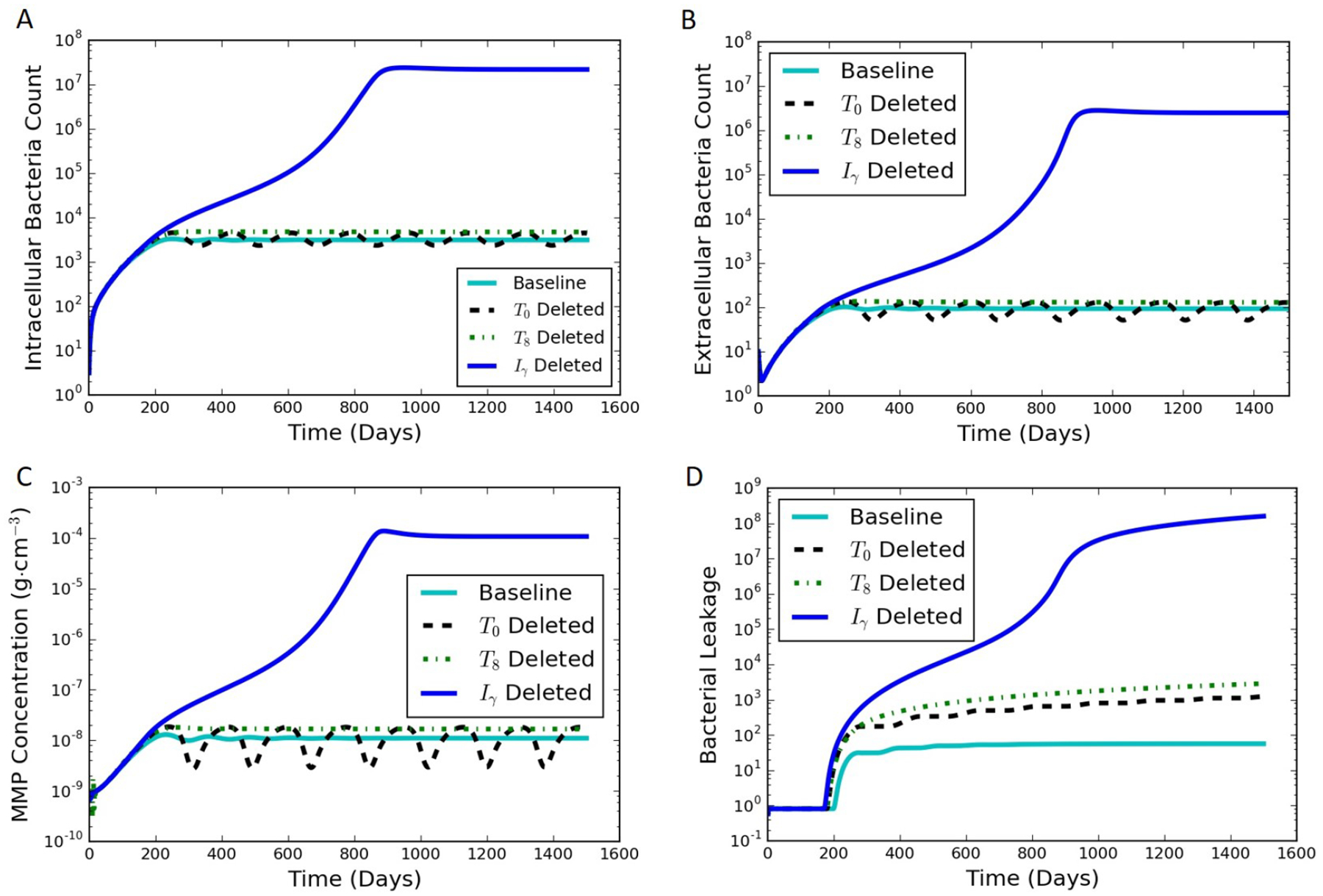
Simulation results of the immune system pertubation experiment where the differential equation corresponding to each of the species shown is set to be zero from day 0, one at a time while the other species follow the otherwise unmodified model equations. Perturbed responses are shown for *T*_0_, *T*_8_, and *I*_*γ*_ (dashed, dotted, and dark solid lines, respectively) The baseline is considered to be a steady latent state (light solid line). (**A**) intracellular bacterial count vs. time; (**B**) extracellular bacterial count vs. time; (**C**) MMP concentration vs. time; (**D**) cumulative bacterial leakage vs. time.

An in silico experiment was carried out using the model to examine the effect of perturbing the immune system through the loss of the single immune system components, such as through gene deletion or pharmaceutical interventions. This was conducted by setting the differential equation corresponding to a specific cytokine or cell type to be equal to zero for all time after an initial condition of zero, representing a synthetic suppression of the production of that cell type or cytokine. The rest of the model equations were left unchanged. When starting from the initial conditions and parameters for the typical latent case, one of four results can occur when a specific component of the immune system is not produced during a sustained perturbation of the typical immune response: (1) active infection; (2) formation of a significantly leaking granuloma; (3) a periodic switching between latent and leaking states; and (4) latent infection with little or no leaking. Table 3 summarizes results of the immune system pertubation experiment, and Figure 7 contains results of the immune system pertubation that showcase representative active, leaking, periodic switching, and latent results. The baseline case shown for comparison was the case discussed in Section 3.1 and shown in Figure 4.

Four species resulted in the active state when they were suppressed: *I*_*γ*_, *T*_1_, *F*_*α*_, and *T*_*C*_. The corresponding intracellular, extracellular and bacterial leakage counts for *I*_*γ*_, *T*_1_, and *F*_*α*_ increased to the order of 10^8^ (Figure 7A,B,D for *I*_*γ*_), demonstrating an active infection. The results for *T*_*c*_ suppression (not shown) quickly exploded the bacterial and infected macrophages count and never stabilized as in the other three cases.

Eliminating either *T*_8_ (Figure 4) or *M*_*A*_ (not shown because of the similarity to *T*_8_) created a leaking granuloma state characterized by a non-oscillating substantial increase in bacterial leakage (on the order of 10^3^). The non-oscillating leakage indicated that the collagen was not able to re-form and control the infection.

We have termed an intermediate state between latent and leaking as “periodic switching” because of sustained oscillations. Eliminating *T*_0_, *T*_80_, or *I*_12_ leads to oscillations in bacterial counts as well as the MMP concentration (Figure 7 for *T*_0_). The constant amplitude of oscillations in the intracellular and extracellular bacterial count and MMP levels suggest that the system was continuously oscillating between two states. For every drop in the MMP levels, the bacterial leakage count stayed steady marking a non-leaking state (flat zone in the stair step pattern of the bacterial leakage curve in Figure 7D). In contrast, as the MMP peaked in every oscillation, it caused a certain constant number of the extracellular bacteria to leak. This led to a corresponding increase in the cumulative bacterial leakage at that point, thus marking a leaking state. The amplitudes of the oscillations for *T*_0_ and *T*_80_ were similar, while *I*_12_ had a larger amplitude of oscillations in the range of 20–200 *B*_*E*_ (similar maximum as the other cases but with a lower minimum). The resulting magnitude of bacterial leakage was the same for all three cases, but the duration of the latent periods was longer for *I*_12_ (not shown).

The simulations for suppressing *T*_2_, *I*_4_, and *I*_10_ resulted in latent cases (not shown). The results for *T*_2_ suppression were nearly indistinguishable from the baseline case. The bacterial leakage for *I*_4_ was slightly lower than that for the baseline case. The results for *I*_10_ suppression showed oscillations in *B*_*I*_ and *B*_*E*_ lower than the baseline levels and zero leakage.

### 3.5. In Silico HIV Co-Infection Experiment via T Cell Depletion

**Figure 8.**
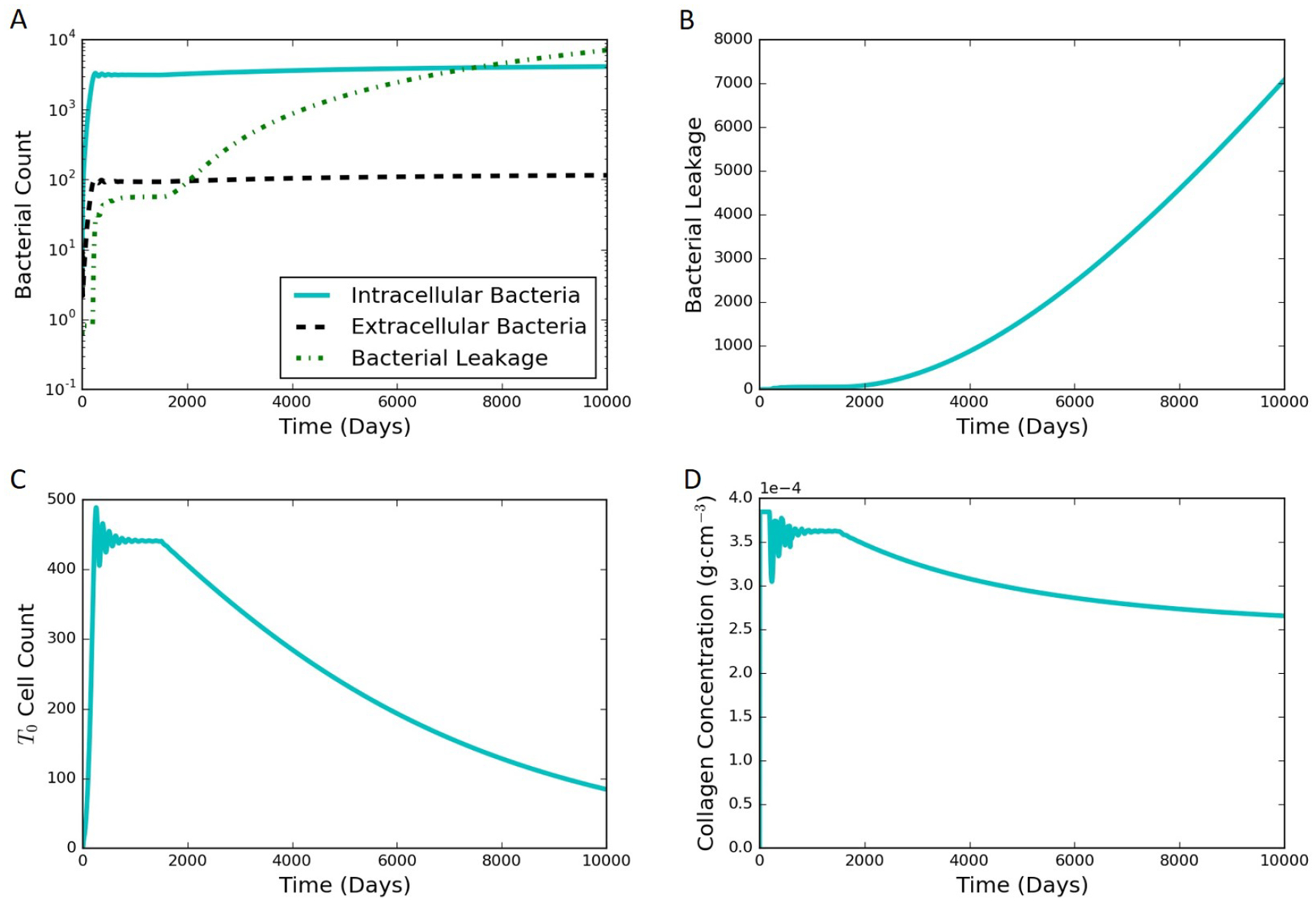
Simulation results for the in silico HIV co-infection experiment via *T*_0_ cell depletion after 1500 days. The depletion results in a leaking state. (**A**) bacterial populations and cumulative bacterial leakage (log scale) vs. time; (**B**) cumulative bacterial leakage (linear scale) vs. time; (**C**) *T*_0_ cell count (linear scale) vs. time; (**D**) concentration of collagen (linear scale) vs. time.

**Figure 9.**
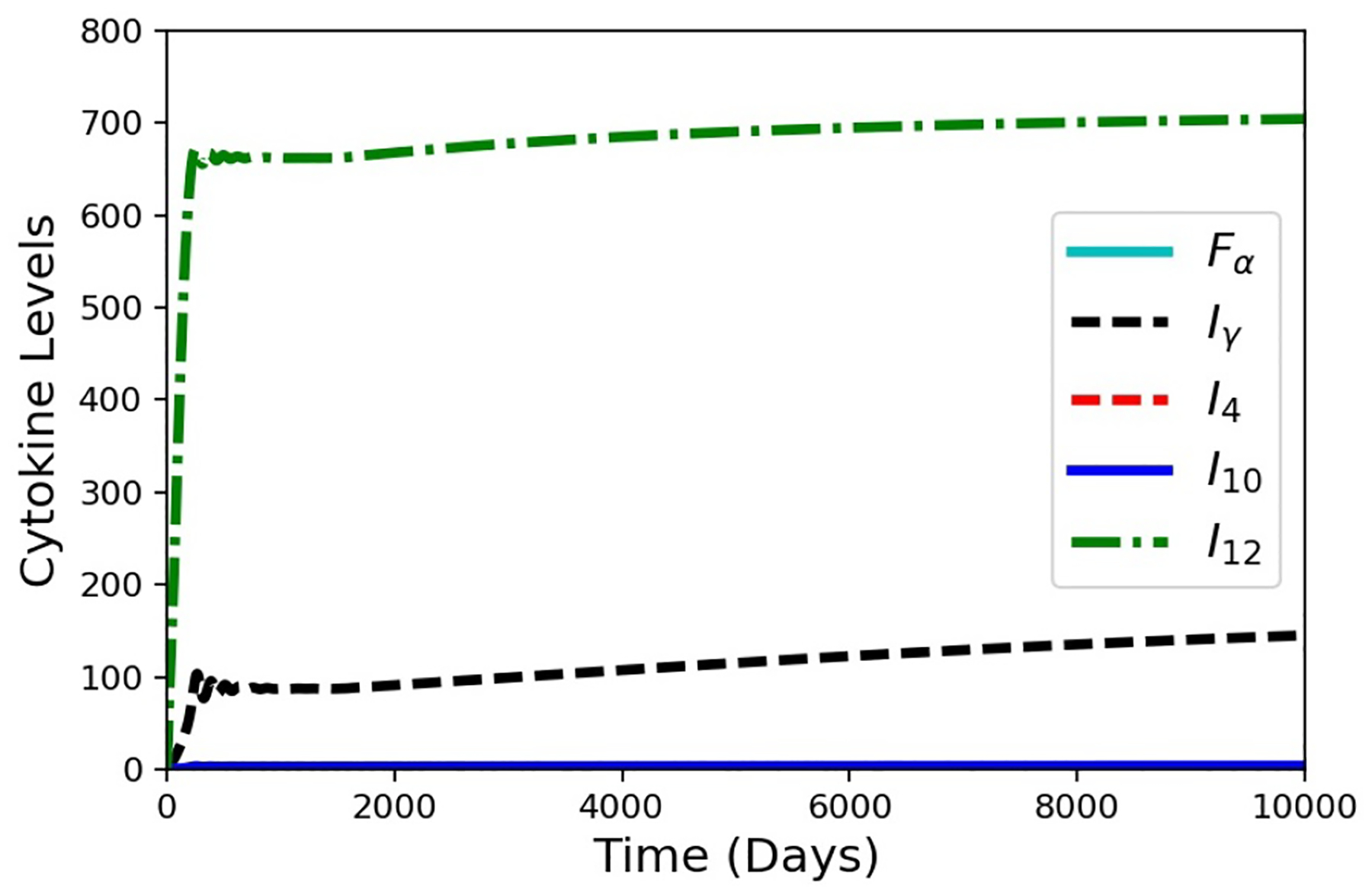
Simulation results for the cytokines from the in silico HIV co-infection experiment via *T*_0_ cell depletion after 1500 days. The depletion results in increasing cytokine levels, except for *I*_4_. The values for *F*_*α*_, *I*_10_, and *I*_4_ are all small.

HIV co-infection is known to increase the risk of progression from latent TB to active disease. When a patient is infected with HIV, a major immunological effect is reduction in CD4+ T cells. To model a patient with latent TB becoming co-infected with HIV, an in silico experiment to deplete precursor *T*_0_ cells was performed (Figure 8). The model was run for 1500 days with the baseline case and no changes to the model to establish a latent condition. At 1500 days, parameter *α*_1*a*_ was set to zero, and parameters *α*_2_ and *sr*_1*b*_ were gradually reduced by an exponentially decaying function for the next 1000 days. All three of these parameters are associated with the number of *T*_0_ cells present within the granuloma (smaller values of the parameters correspond to reduced production and recruitment of T cells). The simulation results show that the system tried to stabilize to until 1500 days (Figure 8) with damping oscillations in *T*_0_ at latency. Shortly after day 1500, there was drop in *T*_0_ cell count for the co-infection. This changed the levels of the cytokines and infected macrophages that affected MMP and collagen concentrations, eventually leading to an increase in bacterial leakage. These results show that a reduction in *T*_0_ cells simulating HIV co-infection after development of latent TB can indeed trigger degradation of collagen and induce a leaking granuloma. The simulation results are consistent with an experimental study in mice co-infected with HIV and Mtb that showed increased mycobacterial burden and dissemination, loss of granuloma structure, and increase progression of TB-disease when the HIV co-infection was present [27]. Another experimental work showed that TB granulomas within HIV-positive expressed more IFN-*γ*, TNF-*α*, IL-4, and IL-12 than granulomas from HIV-negative individual [28,29]. Our cytokine results for the HIV co-infection simulation yield gradually increasing levels of IFN-*γ*, TNF-*α*, and IL-12 and small decreasing levels of IL-4 (Figure 9).

## 4. Conclusions

In this study, we extended a model for the immune response to Mtb by adding new equations describing the dynamics of MMP upregulation, collagen degradation, and bacterial leakage. These new equations are able to produce a leaking regime and periodic switching between leaking and non-leaking in addition to the active and latent states that could be modeled with the immune response model. The simulations’ results in Section 3 show how MMP–collagen interactions play a significant role in the creation of leaking granulomas, leading to spreading of the infection. Varying the parameter *k*_*c*_ that governs the degradation rate of collagen by MMP had major consequences on the transition from the latent state to the leaking granuloma state. Using this model, we were also able to assess the effects of perturbing the immune response to suppress the responses of various cells and cytokines on the granuloma state in the long-term and the effects of depletion of *T*_0_ cells to simulate HIV co-infection. The model fills a gap in the mathematical modeling of the processes of granuloma activation after latency. The model opens up new directions for computational and experimental studies related to the long-term prognosis of patients with latent TB such as effects of co-infections or vaccines and for further exploration of the dynamics of granuloma activation after latency to improve the understanding of TB disease progression and treatments.

## Supplementary Material

supplementary materials

## Figures and Tables

**Table 1.** Parameters in the tuberculosis (TB) granuloma activation model (MMP: matrix metalloproteinase; *M*_*I*_: infected macrophages; TNF: tumor necrosis factor).

Parameter	Description	Value	Units
*α*_MMP_	Rate constant for production of MMP by stromal cells	5.75 × 10^−10^	$\text{g}\cdot {\text{cm}}^{-3}\cdot M_{I}^{-1}$
*β*_MMP_	Rate constant for production of MMP by *M*_*I*_	4.44 × 10^−10^	$\text{g}\cdot {\text{cm}}^{-3}\cdot M_{I}^{-1}$
*s*_MMP_	Half-sat constant of the effect of TNF-*α* on TNF-*α* dependent MMP production	2 × 10^−1^	pg · cm^−3^
*μ*_MMP_	Half life of MMP	4.5	day^−1^
*sr*_MMP_	Constant recruitment rate of MMPs	3.2 × 10^−9^	g · cm^−3^ · day^−1^
*sr*_*C*_	Constant recruitment rate of collagen	1.21 × 10^−4^	g · cm^−3^ · day^−1^
*k*_*C*_	Rate constant of collagen degradation	1.41 × 10^5^	day^−1^
*k*_*M*_	Half-sat constant of the effect of collagen on collagen degradation	4.289 × 10^−3^	g · cm^−3^
*k*_*L*_	Rate of bacterial leakage at zero collagen	0.1	*B*_*E*_ · day^−1^
*s*_*L*_	Half-sat constant on the effect of collagen depletion on bacterial leakage	2 × 10^−4^	g · cm^−3^
*C*_*La*_	Concentration of collagen at latency	3.62 × 10^−4^	g · cm^−3^

**Table 2.** Initial conditions for each species in the combined immune response and TB granuloma activation model (TNF: tumor necrosis factor; IFN: interferon; IL: interleukin).

Species	Description	Initial Value	Units
*M*_*R*_	Resting macrophage count	3.0 × 10^5^	Count
*M*_*I*_	Infected macrophage count	0	Count
*M*_*A*_	Activated macrophage count	0	Count
*T*_0_	Th0 cells count	0	Count
*T*_1_	Th1 cells count	0	Count
*T*_2_	Th2 cells count	0	Count
*T*_80_	T80 cells count	0	Count
*T*_8_	T8 cells count	0	Count
*T*_*c*_	TC cells count	0	Count
*B*_*I*_	Intracellular bacteria	0	Count
*B*_*E*_	Extracellular bacteria introduced by infection	10	Count
*B*_*E,IR*_	Extracellular bacteria generated during immune response to infection	0	Count
*B*_*E,L*_	Leaking extracellular bacteria	0	Count
*F*_*α*_	TNF-*α* concentration	0	pg · mL^−1^
*I*_*γ*_	IFN-*γ* concentration	0	pg · mL^−1^
*I*_4_	IL-4 concentration	0	pg · mL^−1^
*I*_10_	IL-10 concentration	0	pg · mL^−1^
*I*_12_	IL-12 concentration	0	pg · mL^−1^
*C*	Collagen concentration	0	g · g^−3^
*MMP*	MMP concentration	7.11 × 10^−10^	g · g^−3^

**Table 3.** Summary of in silico immune system pertubation results.

Species Suppressed in the Simulation	Notation	Resulting State
IFN-*γ*	*I*_*γ*_	Active
CD4+ Th1 cells	*T*_1_	Active
TNF-*α*	*F*_*α*_	Active
CD8+ Tc cells	*T*_*c*_	Active and highly unstable
CD8+ T8 cells	*T*_8_	Leaking
Activated macrophages	*M*_*A*_	Leaking
CD4+ Th0 cells	*T*_0_	Periodic switching
CD8+ T80 cells	*T*_80_	Periodic switching
IL-12	*I*_12_	Periodic Switching
CD4+ Th2 cells	*T*_2_	Latent
IL-4	*I*_4_	Latent
IL-10	*I*_10_	Latent
