# Supplementary material for: Mathematical Modeling of Tuberculosis Granuloma Activation
Source: Processes (Basel). Author manuscript; Available in PMC 2022 Jan 5. (PMC8730292; doi:10.3390/pr5040079)
Supplement: supplementary materials [file NIHMS1764464-supplement-supplementary_materials.pdf]

Supplementary Materials: Mathematical Modeling of Tuberculosis Granuloma Activation

Steve M. Ruggiero, Minu R. Pilvankar, and Ashlee N. Ford Versypt

The equations (1)–(4) for TB granuloma activation in the paper are combined with the immune response model developed in [1], which included the following equations (S1)–(S16) to track the populations of

- macrophages
  - resting macrophages, M_R
  - infected macrophages, M_I
  - activated macrophages, M_A
- CD4+ T cells
  - Th0 cells, T_0
  - Th1 cells, T_1
  - Th2 cells, T_2
- CD8+ T cells
  - T80 cells, T_{80}
  - T8 cells, T_8
  - TC cells, T_c
- bacteria
  - intracellular bacteria contained inside of infected macrophages, B_I
  - extracellular bacteria located inside the granuloma but outside of any cells, B_E (note that in [1] the only source of B_E is the $B_{E,IR}$ contribution from the immune response, whereas the model developed in the present work also includes a contribution $B_{E,L}$ from the bacterial leakage)
  - total bacteria, $B_T = B_E + B_I$

and concentrations of

- cytokines
  - TNF-α, F_a
  - IFN-γ, I_γ
  - IL-4, I_4
  - IL-10, I_{10}
  - IL-12, I_{12}

with the parameters defined in Table S1:

$$\begin{aligned}\frac{dM_R}{dt} &= sr_M + \alpha_{4A}(M_A + \omega_2 M_I) + sr_{4B}\frac{F_a}{F_a + f_8 I_{10} + s_{4b}} \\ &\quad - k_2 M_R \frac{B_E}{B_E + c_9} - k_3 M_R \frac{I_\gamma}{I_\gamma + f_1 I_4 + s_1}\frac{B_T + \beta F_\alpha}{B_T + \beta F_\alpha + c_8} - \mu_{MR} M_R\end{aligned} \tag{S1}$$

$$\begin{aligned}\frac{dM_I}{dt} &= k_2 M_R \frac{B_E}{B_E + c_9} - k_{17} M_I \frac{B_I^2}{B_I^2 + (NM_I)^2} \\ &\quad - k_{14A} M_I \frac{(T_c + \omega_3 T_1)/M_I}{(T_c + \omega_3 T_1)/M_I + c_4} - k_{14B} M_I \frac{F_\alpha}{F_\alpha + f_9 I_{10} + s_{4B}} \\ &\quad - k_{52} M_I \frac{(T_c(T_1/(T_1 + c_{T1})) + \omega_1 T_1)/M_I}{(T_c(T_1/(T_1 + c_{T1})) + \omega_1 T_1)/M_I + c_{52}} - \mu_{MI} M_I\end{aligned} \tag{S2}$$

$$\frac{dM_A}{dt} = k_3 M_R \frac{I_\gamma}{I_\gamma + f_1 I_4 + s_1} \frac{B_T + \beta F_\alpha}{B_T + \beta F_\alpha + c_8} - k_4 M_A \frac{I_{10}}{I_{10} + s_8} - \mu_{MA} M_A \tag{S3}$$

$$\begin{aligned} \frac{dT_0}{dt} &= \alpha_{1A}(M_A + \omega_2 M_I) + sr_{1B} \frac{F_a}{F_a + f_8 I_{10} + s_{4b2}} + \alpha_2 T_0 \frac{M_A}{M_A + c_{15}} \\ &\quad - k_6 I_{12} T_0 \frac{I_\gamma}{I_\gamma + (f_1 I_4 + f_7 I_{10}) + s_1} - k_7 T_0 \frac{I_4}{I_4 + f_2 I_\gamma + s_2} - \mu_{T0} T_0 \end{aligned} \tag{S4}$$

$$\begin{aligned} \frac{dT_1}{dt} &= \alpha_{3A}(M_A + \omega_2 M_I) + sr_{3B} \frac{F_a}{F_a + f_8 I_{10} + s_{4b1}} \\ &\quad + k_6 I_{12} T_0 \frac{I_\gamma}{I_\gamma + (f_1 I_4 + f_7 I_{10}) + s_1} - \mu_{T\gamma} \frac{I_\gamma}{I_\gamma + c} T_1 M_A - \mu_{T1} T_1 \end{aligned} \tag{S5}$$

$$\begin{aligned} \frac{dT_2}{dt} &= \alpha_{3A2}(M_A + \omega_2 M_I) + sr_{3B2} \frac{F_a}{F_a + f_8 I_{10} + s_{4b1}} \\ &\quad + k_7 T_0 \frac{I_4}{I_4 + f_2 I_\gamma + s_2} - \mu_{T2} T_2 \end{aligned} \tag{S6}$$

$$\begin{aligned} \frac{dT_{80}}{dt} &= \alpha_{1A}(M_A + \omega_2 M_I) + sr_{1B} \frac{F_a}{F_a + f_8 I_{10} + s_{4b2}} + \alpha_2 T_{80} \frac{M_A}{M_A + c_{15}} \\ &\quad - k_6 I_{12} T_{80} \frac{I_\gamma}{I_\gamma + (f_1 I_4 + f_7 I_{10}) + s_1} - \mu_{T80} T_{80} \end{aligned} \tag{S7}$$

$$\begin{aligned} \frac{dT_8}{dt} &= m\alpha_{3Ac}(M_A + \omega_2 M_I) + msr_{3Bc} \frac{F_a}{F_a + f_8 I_{10} + s_{4b1}} \\ &\quad + mk_6 I_{12} T_{80} \frac{I_\gamma}{I_\gamma + (f_1 I_4 + f_7 I_{10}) + s_1} - \mu_{Tc\gamma} \frac{I_\gamma}{I_\gamma + c_c} T_8 M_A - \mu_{T8} T_8 \end{aligned} \tag{S8}$$

$$\begin{aligned} \frac{dT_c}{dt} &= m\alpha_{3Ac}(M_A + \omega_2 M_I) + msr_{3Bc} \frac{F_a}{F_a + f_8 I_{10} + s_{4b1}} \\ &\quad + mk_6 I_{12} T_{80} \frac{I_\gamma}{I_\gamma + (f_1 I_4 + f_7 I_{10}) + s_1} - \mu_{Tc\gamma} \frac{I_\gamma}{I_\gamma + c_c} T_c M_A - \mu_{Tc} T_c \end{aligned} \tag{S9}$$

$$\begin{aligned} \frac{dF_\alpha}{dt} &= \alpha_{30} M_I + \alpha_{30} M_A \frac{I_\gamma + \beta_2 B_T}{I_\gamma + \beta_2 B_T + (f_1 I_4 + f_7 I_{10}) + s_{10}} \\ &\quad + \alpha_{32} T_1 + \alpha_{33}(T_c + T_8) - \mu_{F\alpha} F_\alpha \end{aligned} \tag{S10}$$

$$\begin{aligned} \frac{dI_\gamma}{dt} &= s_g \frac{B_T}{B_T + c_{10}} \frac{I_{12}}{I_{12} + s_7} + \alpha_{5A} T_1 \frac{M_A}{M_A + c_{5A}} + \alpha_{5B} T_8 \frac{M_A}{M_A + c_{5B}} \\ &\quad + \alpha_{5c} M_I + \alpha_7 T_0 \frac{I_{12}}{I_{12} + f_4 I_{10} + s_4} + \alpha_7 T_{80} \frac{I_{12}}{I_{12} + f_4 I_{10} + s_4} - \mu_{I\gamma} I_\gamma \end{aligned} \tag{S11}$$

$$\frac{dI_4}{dt} = \alpha_{11} T_0 + \alpha_{12} T_2 - \mu_{I4} I_4 \tag{S12}$$

$$\frac{dI_{10}}{dt} = \delta_7 M_A \frac{s_6}{I_{10} + f_6 I_\gamma + s_6} + \alpha_{16} T_1 + \alpha_{17} T_2 + \alpha_{18}(T_8 + T_c) - \mu_{I10} I_{10} \tag{S13}$$

$$\frac{dI_{12}}{dt} = s_{12} \frac{B_T}{B_T + c_{230}} + \alpha_{23} M_R \frac{B_T}{B_T + c_{23}} + \alpha_8 M_A \frac{s}{s + I_{10}} - \mu_{I12} I_{12} \tag{S14}$$

$$\begin{aligned}\frac{dB_I}{dt} &= \alpha_{19} B_I \left(1 - \frac{B_I^2}{B_I^2 + (NM_I)^2}\right) + k_2 \frac{N}{2} M_R \frac{B_E}{B_E + c_9} \\ &- k_{17} NM_I \frac{B_I^2}{B_I^2 + (NM_I)^2} - k_{14A} NM_I \frac{(T_c + \omega_3 T_1)/M_I}{(T_c + \omega_3 T_1)/M_I + c_4} \\ &- k_{14B} NM_I \frac{F_\alpha}{F_\alpha + f_9 I_{10} + s_{4b}} \\ &- k_{52} NM_I \frac{(T_c(T_1/(T_1 + c_{T1})) + \omega_1 T_1)/M_I}{(T_c(T_1/(T_1 + c_{T1})) + \omega_1 T_1)/M_I + c_{52}} - \mu_I B_I\end{aligned} \tag{S15}$$

$$\begin{aligned}\frac{dB_E}{dt} = \frac{dB_{E,IR}}{dt} &= \alpha_{20} B_E + \mu_I B_I - k_{15} M_A B_E - k_{18} M_R B_E + k_{17} NM_I \frac{B_I^2}{B_I^2 + (NM_I)^2} \\ &- k_2 \frac{N}{2} M_R \frac{B_E}{B_E + c_9} + k_{14A} NN_{fracc} M_I \frac{(T_c + \omega_3 T_1)/M_I}{(T_c + \omega_3 T_1)/M_I + c_4} \\ &+ k_{14B} NN_{fraca} M_I \frac{F_\alpha}{F_\alpha + f_9 I_{10} + s_{4b}}\end{aligned} \tag{S16}$$

For detailed descriptions of the derivation of (S1)–(S16), see [1] and [2]. Diagrams of the kinetic processes in the immune response model for macrophages, T cells, and bacteria are illustrated in [2].

Table S1. Parameters used in immune response model from [1]

Parameter	Description	Value	Units
α_{5a}	Production of I_γ by T_1	50	$pg \cdot T_1^{-1} \cdot day^{-1}$
α_{30}	Production of F_α by M_I	3×10^{-3}	$pg \cdot ml^{-1} \cdot M_I^{-1} \cdot day^{-1}$
α_{5c}	Production of I_γ by M_I	0.03	$pg \cdot ml^{-1} \cdot M_I^{-1}$
α_{4a}	Recruitment of M_R	5×10^{-3}	day^{-1}
α_{23}	Production of I_{12} by M_R	2×10^{-4}	$pg \cdot ml^{-1} \cdot M_R^{-1}$
α_{5b}	Production of I_γ by T_8	50	$pg \cdot T_8^{-1} \cdot day^{-1}$
α_{18}	Production of I_{10} by T_c and T_8	0.02	$pg \cdot CD8^{-1} \cdot day^{-1}$
α_{3a2}	Recruitment of T_2 by chemokines	1×10^{-3}	day^{-1}
α_{3ac}	Recruitment of T_c and T_8	3×10^{-3}	day^{-1}
α_{31}	Production of F_α by M_A	4×10^{-3}	$pg \cdot ml^{-1} \cdot M_A^{-1} \cdot day^{-1}$
α_{1a}	Recruitment of T_0	4×10^{-3}	day^{-1}
α_{32}	Production of F_α by T_1	8.16×10^{-4}	$pg \cdot ml^{-1} \cdot T_1^{-1} \cdot day^{-1}$
α_{33}	Production of F_α by T_8	6×10^{-5}	$pg \cdot ml^{-1} \cdot T_8^{-1} \cdot day^{-1}$
α_{3a}	Recruitment of T_1	5×10^{-3}	day^{-1}
sr_{3b2}	F_α dependent recruitment of T_2	1×10^3	day^{-1}
sr_{4b}	F_α dependent recruitment of M_R	2×10^4	$M_R \cdot day^{-1}$
sr_{1b}	F_α dependent recruitment of T_0	2×10^5	$T_0 \cdot day^{-1}$

Table S1. Parameters used in immune response model from [1]

Parameter	Description	Value	Units
sr_{3b}	F_α dependent recruitment of T_1	2×10^4	$T_0 \cdot \text{day}^{-1}$
sr_{3bc}	F_α dependent recruitment of T_c and T_8	8×10^4	$T \cdot \text{day}^{-1}$
f_9	Ratio adjustment of F_α and I_{10}	50	-
f_7	Effect of I_{10} on I_γ induced T_0 differentiation to T_1	1	-
f_8	Ratio adjustment of F_α and I_{10} on M_R recruitment	1	-
s_{4b1}	Half-sat constant of F_α on T_1 recruitment	165	$\text{pg} \cdot \text{ml}^{-1}$
s_{4b2}	Half-sat constant of F_α on T_0 recruitment	450	$\text{pg} \cdot \text{ml}^{-1}$
s_{4b}	Half-sat constant of F_α on M_R recruitment	200	$\text{pg} \cdot \text{ml}^{-1} \cdot \text{day}^{-1}$
β_2	Scaling factor of B_T for F_α production by M_A	10^{-3}	-
β	Scaling factor of F_α for M_R activation to M_A	100	$B_T \cdot \text{pg}^{-1}$
c	Half-sat constant of I_γ on T_1 death	1.1×10^3	$\text{pg} \cdot \text{ml}^{-1}$
c_c	Half-sat constant of I_γ on T_c and T_8 death	550	$\text{pg} \cdot \text{ml}^{-1}$
c_{52}	Half-sat constant of T_1 on T_c cytotoxicity	50	T_C
c_{T1}	Half-sat constant of T_c on T-cell induced M_I killing	10	T_1
c_{5a}	Half-sat constant of M_A on I_γ production by T_1	7×10^3	$M_A \cdot \text{ml}^{-1}$
c_T	Half-sat constant of B_T on F_α production by T_1 and T_8	1×10^4	B_T
c_{5b}	Half-sat constant of M_A on I_γ production T_8	7×10^3	$M_A \cdot \text{ml}^{-1}$
c_{230}	Half-sat constant of B_T on I_{12} production by dendritic cells	1×10^3	$B_T \cdot \text{ml}$
c_{23}	Half-sat constant of B_T on I_{12} production by M_R	5×10^3	$B_T \cdot \text{ml}$
c_4	Half-sat constant of $\frac{T_c+T_1}{M_I}$ on M_I apoptosis	40	$T \cdot M_I^{-1}$
ω_3	Max percent contribution of T_1 to Fas-FasL apoptosis of M_I	0.4	-
ω_2	Max percent contribution of M_I produced cytokines to M_R recruitment	0.15	-
ω_1	Max percent contribution of T_1 to cytotoxicity	0.5	-
m	Percent overlap of T_c and T_8	0.6	-
$\mu_{T\gamma}$	Rate of I_γ induced apoptosis of T_1	1×10^{-4}	$M_A^{-1} \cdot \text{day}^{-1}$
$\mu_{Tc\gamma}$	Rate of I_γ induced apoptosis of T_c and T_8	1×10^{-4}	$M_A^{-1} \cdot \text{day}^{-1}$
μ_{T8}	Death rate of T_8	0.33	day^{-1}
μ_{Tc}	Death rate of T_c	0.33	day^{-1}
μ_{T80}	Death rate of T_{80}	0.33	day^{-1}
μ_I	B_I turnover to B_E, primarily due to M_I death	4×10^{-3}	day^{-1}
μ_{TNF}	Decay rate of F_α	1.11	day^{-1}
k_{14a}	Apoptosis of M_I induced by Fas-FasL	0.1	day^{-1}
k_{14b}	Apoptosis of M_I induced by F_α	0.1	day^{-1}
k_{52}	Cytotoxic killing of M_I	0.5	day^{-1}
s_{10}	Half-sat constant of I_γ on F_α production by M_A	80	$\text{pg} \cdot \text{ml}^{-1}$
s_{12}	Production of I_{12} by dendritic cells	1×10^3	$\text{pg} \cdot \text{ml}^{-1} \cdot \text{day}^{-1}$
s	Downregulation by I_{10} on I_{12} production by M_A	10	$\text{pg} \cdot \text{ml}^{-1}$
δ_7	Production of I_{10} by M_A	0.01	$\text{pg} \cdot \text{ml}^{-1} \cdot M_A^{-1}$
N_{fraca}	Average number of bacteria released from M_I apoptosis by F_α	0.5	-
N_{fracc}	Average number of bacteria released from M_I apoptosis by Fas-FasL	0.1	-

Table S1. Parameters used in immune response model from [1]

Parameter	Description	Value	Units
α_{20}	Growth rate of B_E	0.05	day^{-1}
α_{19}	Growth rate of B_I	0.4	day^{-1}
α_{12}	Production of I_4 by T_2	1×10^{-3}	$pg \cdot T_2^{-1} \cdot day^{-1}$
α_{11}	Production of I_4 by T_0	5×10^{-4}	$pg \cdot T_0^{-1} \cdot day^{-1}$
α_{17}	Production of I_{10} by T_2	0.06	$pg \cdot T_2^{-1} \cdot day^{-1}$
α_8	Production of I_{12} by M_A	8×10^{-4}	$pg \cdot M_A^{-1} \cdot day^{-1}$
α_7	Production of I_γ by T_0	0.03	$pg \cdot ml^{-1} \cdot T_0^{-1}$
α_{16}	Production of I_{10} by T_1	2×10^{-3}	$pg \cdot T_1^{-1} \cdot day^{-1}$
α_2	Max growth rate of T_0	5×10^{-3}	day^{-1}
sr_m	Recruitment rate of M_R	1×10^3	$M_R \cdot day^{-1}$
f_6	Ratio adjustment of I_{10} and I_γ on I_{10} production	2.5×10^{-2}	-
f_4	Ratio adjustment of I_{10} and I_{12} on I_γ production	2	-
f_2	Ratio adjustment of I_γ and I_4 on T_0 differentiation to T_2	1	-
f_1	Ratio adjustment of I_4 and I_γ	200	-
s_2	Half-sat constant of I_4 on T_0 differentiation to T_2	5	$pg \cdot ml^{-1}$
s_6	Half-sat constant of I_{10} self-inhibition in production by M_A	60	$pg \cdot ml^{-1}$
s_4	Half-sat constant of I_{12} on production of I_γ by T_0 and T_{80}	50	$pg \cdot ml^{-1}$
s_7	Half-sat constant of I_{12} on I_γ production by NK cells	40	$pg \cdot ml^{-1}$
s_1	Half-sat constant of I_γ on M_R activation to M_A	7×10^3	$pg \cdot ml^{-1}$
s_8	Half-sat constant of I_{10} on M_A deactivation	1	$pg \cdot ml^{-1}$
c_9	Half-sat constant of B_E on M_R infection to M_I	2×10^6	B_E
c_8	Half-sat constant of B_T on M_R activation to M_A	2×10^5	$B_T \cdot ml^{-1}$
c_{15}	Half-sat constant of M_A on I_γ production by T_1	2×10^5	M_A
c_{10}	Half-sat constant of B_T on I_γ production by NK cells	1×10^3	$B_T \cdot ml^{-1}$
μ_{MR}	Death rate of M_R	3.3×10^{-3}	day^{-1}
μ_{MI}	Death rate of M_I	1.1×10^{-3}	day^{-1}
μ_{MA}	Death rate of M_A	0.07	day^{-1}
$\mu_{I\gamma}$	Decay rate of I_γ	2.16	day^{-1}
μ_{I4}	Decay rate of I_4	2.77	day^{-1}
μ_{I10}	Decay rate of I_{10}	5	day^{-1}
μ_{I12}	Decay rate of I_{12}	1.19	day^{-1}
μ_{T2}	Death rate of T_2	0.33	day^{-1}
μ_{T1}	Death rate of T_1	0.33	day^{-1}
μ_{T0}	Death rate of T_0	0.33	day^{-1}
k_2	Rate of M_R infection to M_I	0.4	day^{-1}
k_3	Rate of M_R activation to M_A	0.05	day^{-1}
k_{17}	Max death rate of M_I due to B_I	0.02	day^{-1}
k_4	Rate of M_A deactivation by I_{10}	0.08	day^{-1}
k_6	Max rate of T_0 differentiation to T_1	5×10^{-3}	$ml \cdot pg^{-1} \cdot day^{-1}$
k_7	Max rate of T_0 differentiation to T_2	0.02	$ml \cdot pg^{-1} \cdot day^{-1}$
k_{18}	Rate of B_E killing by M_R	5×10^{-9}	$ml \cdot M_R^{-1} \cdot day^{-1}$

Table S1. Parameters used in immune response model from [1]

Parameter	Description	Value	Units
k_{15}	Rate of B_E killing by M_A	1.25×10^{-7}	$ml \cdot M_A^{-1} \cdot day^{-1}$
s_g	Production of I_γ by NK cells	100	$pg \cdot ml^{-1} \cdot day^{-1}$
N	B_I carrying capacity of M_I	10	$B_I \cdot M_I$

1. Sud, D.; Bigbee, C.; Flynn, J.L.; Kirschner, D.E. Contribution of CD8+ T cells to control of Mycobacterium tuberculosis infection. *J. Immunol.* **2006**, *176*, 4296-4314.
2. Marino, S.; Sud, D.; Plessner, H.; Lin, P.L.; Chan, J.; Flynn, J.L.; Kirschner, D.E. Differences in reactivation of tuberculosis induced from anti-TNF treatments are based on bioavailability in granulomatous tissue, *PLoS Comput. Biol.* **2007**, *3*, e194.
